# Supplementary material for: Selective Recovery of Palladium (II) from Metallurgical Wastewater Using Thiadiazole-Based Chloromethyl Polystyrene-Modified Adsorbent
Source: Int J Mol Sci. 2022 Oct 12;23(20):12158. doi: 10.3390/ijms232012158 (PMC9603776; doi:10.3390/ijms232012158)
Supplement: Supplementary file 1 [file ijms-23-12158-s001.zip › ijms-1946889-supplementary.pdf]

Supplementary Materials for

Selective Recovery of Palladium (II) from Metallurgical Wastewater Using Thiadiazole-based Chloromethyl Polystyrene-modified Adsorbent

Table S1. The synthetic pathway of 2, 5-bis-polystyrene-1,3,4-thiadiazole (PS-DMTD) and 2,5-bis(benzylthio)-1,3,4-thiadiazole (DTTD).

Structure	Synthetic scheme
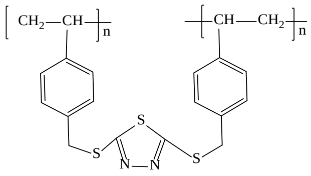 PS-DMTD	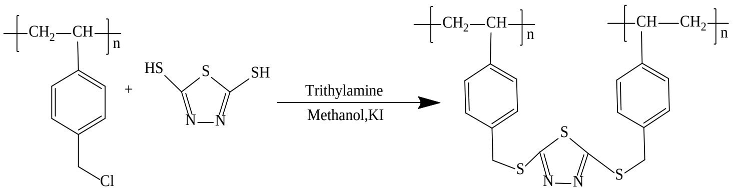
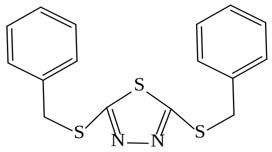 DTTD	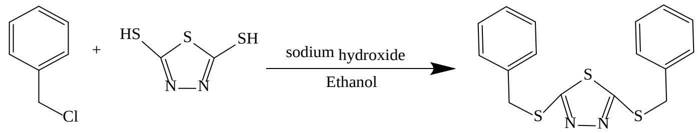

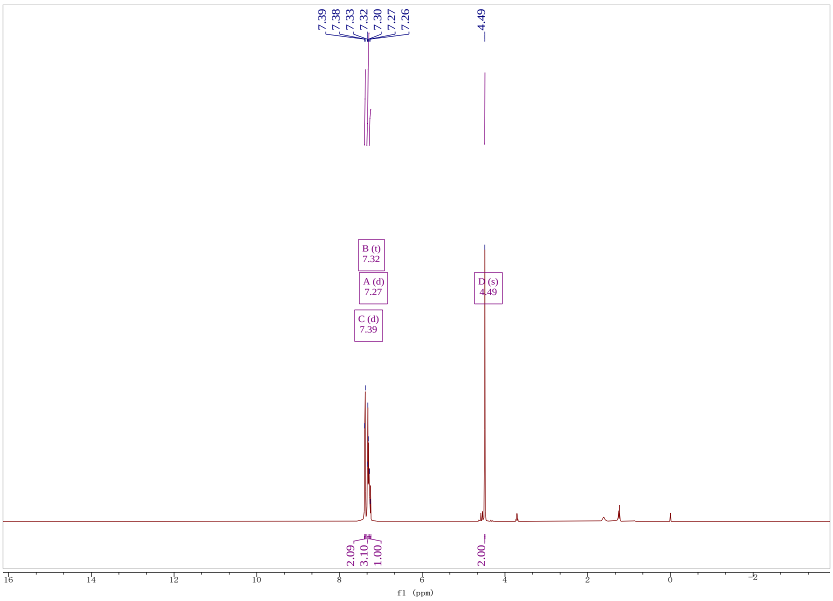

Figure S1. ^{1}H NMR spectrum of DTTD.

^{1}H NMR (500 MHz, Chloroform-d) δ 7.39 (d, J = 7.5 Hz, 4H), 7.32 (t, J = 7.2 Hz, 4H), 7.27 (d, J = 7.2 Hz, 2H), 4.49 (s, 4H).

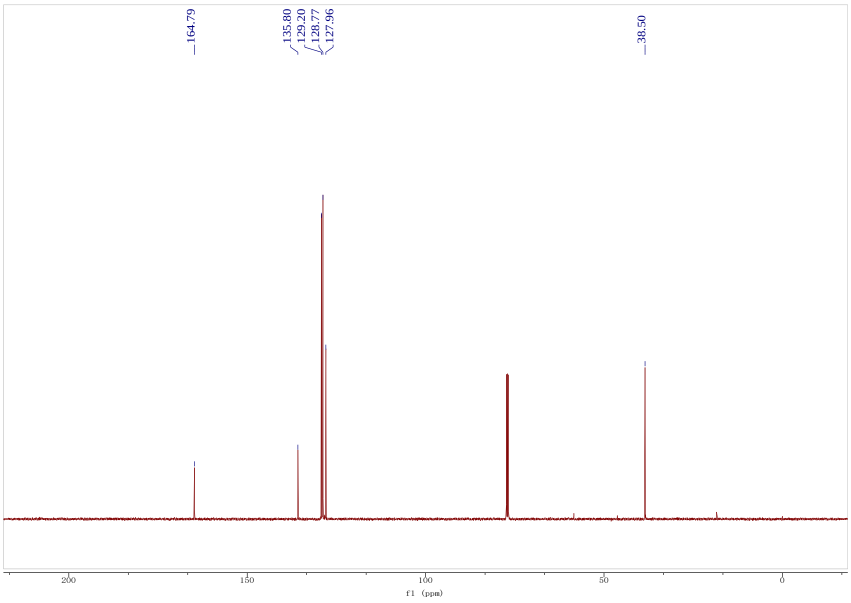

Figure S2. ^{13}C NMR spectrum of DTTD.

^{13}C NMR (125 MHz, Chloroform-d) δ 164.79, 135.80, 129.20, 128.77, 127.96, 38.50.

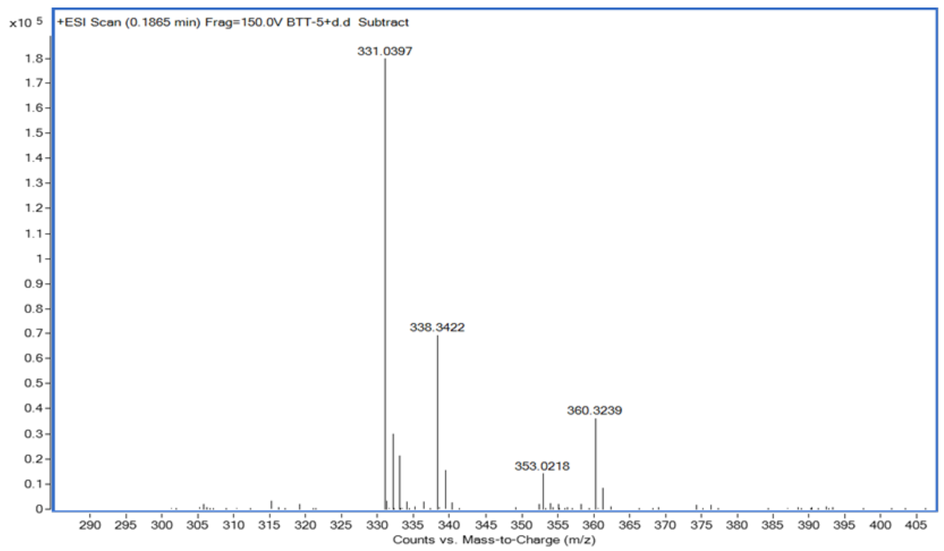

Figure S3. HPLC-MS spectrum of DTTD (ESI scan).

HRMS(ESI)m/s: found: 331.0397 ($M+H^+$); calc: 331.0392 ($M+H^+$).

Table S2. Elemental analysis for PS-Cl and PS-DMTD.

	Elemental analysis		
	**N (%)**	**C (%)**	**H (%)**
PS-Cl	0.458	81.45	7.825
PS-DMTD	2.536	77.56	7.22

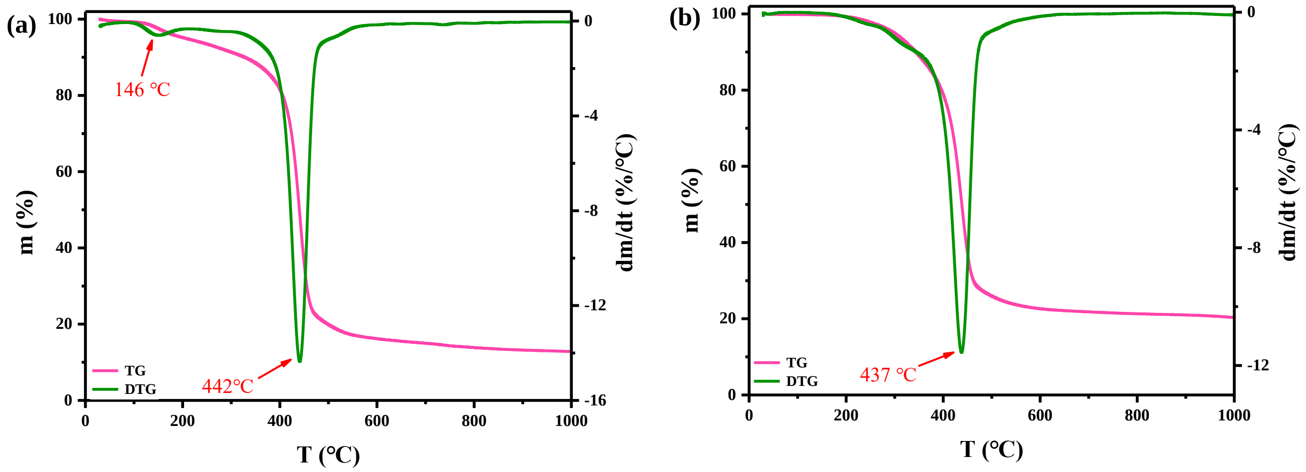

Figure S4. TGA curves of PS-Cl (a), and PS-DMTD (b).

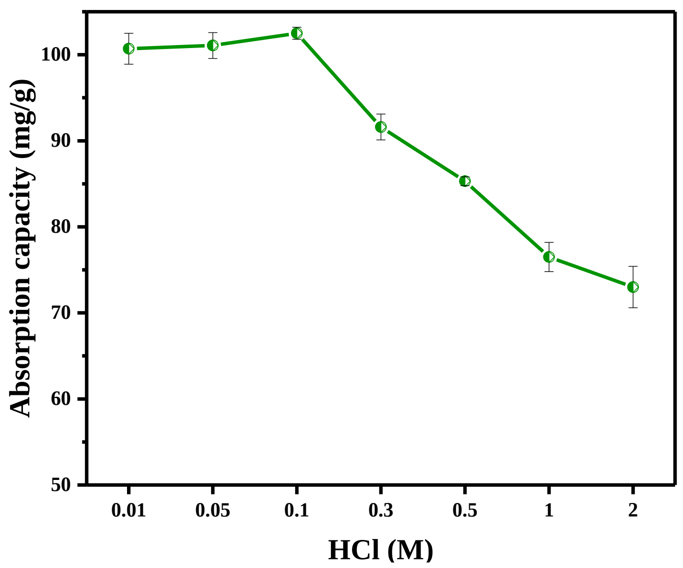

Figure S5. Influence of the concentration of HCl in solution.

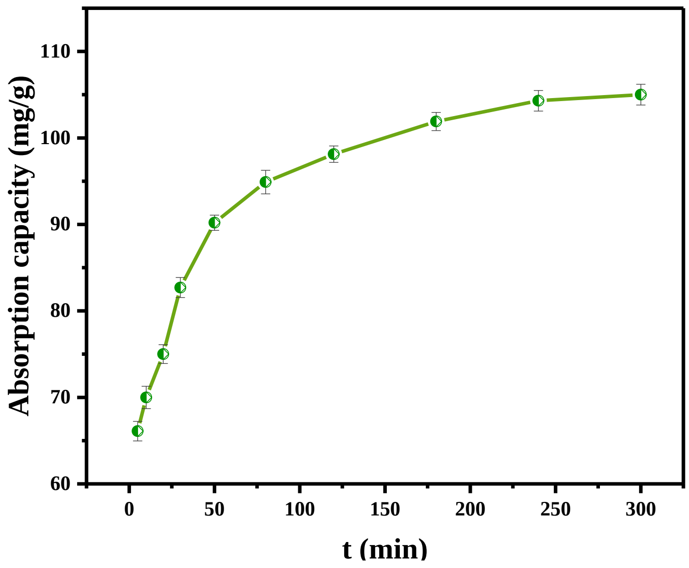

Figure S6. Influence of contact time on adsorption of Pd (II).

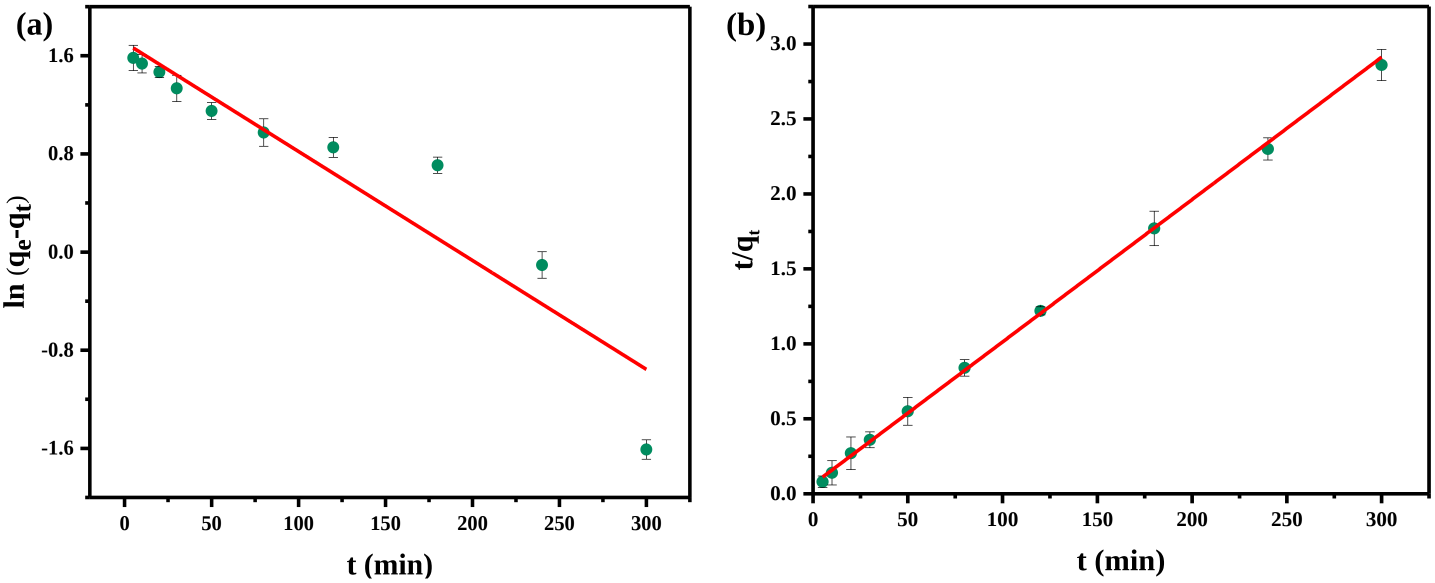

Figure S7. Pseudo-first-order kinetic model fitting curve (a); and pseudo-second-order kinetic model fitting curve (b) of PS-DMTD.

$$R^2_{adj} = 1 - (1 - R^2) \cdot \left(\frac{n-1}{n-p-1}\right) \quad \text{(S1)}$$

$$RSS = \sum_i^n (q_{i,\,exp} - q_{i,\,model})^2 \quad \text{(S2)}$$

$$BIC = nLn\left(\frac{RSS}{n}\right) + pLn\,(n) \quad \text{(S3)}$$

where n is the number of experiments; p is the number of parameters in the fitting model; $q_{i,model}$ is individual theoretical q value predicted by the model; and $q_{i,exp}$ is individual experimental q value.

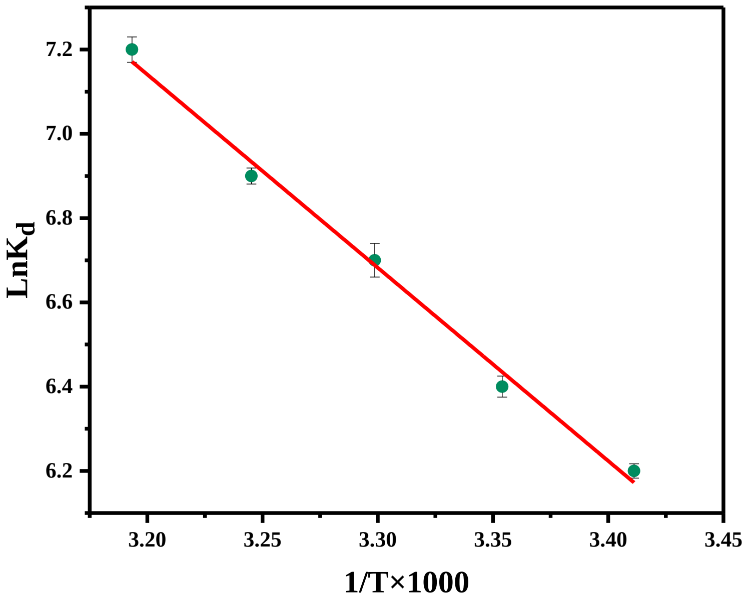

Figure S8. The relationship between ln K_d and 1/T.

Table S3. The composition of the metallurgical wastewater.

Metal ions	The initial concentration (mg/L)
Pd (II)	109.0
Pt (IV)	52.8
Rh (III)	42.6
Ca^{2+}	20.3
Cu^{2+}	390.0
Fe^{3+}	5921.7
Ni^{2+}	685.4
Pb^{2+}	19.0
V^{3+}	7.2
Ti^{4+}	13.7

Table S4. The crystal data of Pd (II)-$DTTD_2Cl_2$.

Parameters	Data
Empirical formula	$C_{32}H_{28}N4S_6Cl_2Pd$
Formula weight	838.24
Temperature	298.15 K
Crystal system	Triclinic
Space group	$P\bar{1}$
Unit cell dimensions	a = 8.4697 (3) Å α = 116.290 (1)°<br>b = 10.3993 (4) Å β = 100.510 (1)°<br>c = 11.2654 (4) Å γ = 91.792 (1)°
Volume	867.59 (6) Å^3
Z	1
Calculated density	1.604 mg/m^3
μ/mm^{-1}	9.35 mm^{-1}
F (000)	424
Crystal size	0.08 × 0.06 × 0.05 mm
Theta range for data collection	8.972 to 133.716°
Limiting indices	-10 < = h < = 10, -12 < = k < = 12, -13 < = l < = 13
Reflections collected	22890
Data/restraints/parameters	3075/0/205
Goodness-of-fit on F^2	1.027
Final R indices [I > 2sigma (I)]	R_1 = 0.0242, wR_2 = 0.0660
R indices (all data)	R_1 = 0.0251, wR_2 = 0.0669
Largest diff. peak and hole	0.61 and -0.39 e Å-3
